# Supplementary material for: Learning curve and surgical outcome of robotic assisted colorectal surgery with ERAS program
Source: Sci Rep. 2022 Nov 29;12:20566. doi: 10.1038/s41598-022-24665-w (PMC9709162; doi:10.1038/s41598-022-24665-w)
Supplement: Supplementary file 1 — Supplementary Information 1. [file 41598_2022_24665_MOESM1_ESM.zip › LSC Raw data-Ñ[▒K/140/H_ñw╝╨░O▒Kñσ.pdf]

正式摘要(電子簽章：000000227293063)

W83 / W83 ☐ 英文標題(注意:無英文姓名-請補病人基本檔!) ☐【讀取】憑證

臺中榮民總醫院

出院病歷摘要

轉入號： 轉出號：

[redacted]

(8)流水編號	
(9)入院日期	110/10/04 (2021) 直腸外科 W83 - 102
(10)轉科日期	
(11)出院日期	110/10/14 (2021) 住院天數計 10 日

診斷

- 入院:Colon Cancer, c T1 N0 M0, Stage I
  [Hx]
  1. HFrEF, FC III, LVEF:24%
  2. Complete AV block s/p PPM insertion on 1996/8/20 (DDDR mode), upgrade to Cardiac resynchronization therapy on 2010/3/5, CRT change generator on 2020/12/21. with generator in pending failure
  3. CAD-TVD s/p PCI + stent on 2013/9, 2013/10
  4. Moderate to severe AR, moderate MR, with severe impair LV function(LVEF 19%) s/p Mitral valve repair (posterior annuloplasty with SJM 31 mm ring) + Aortic valve replacement with tissue valve (SJM porcine valve 25 mm) on 2014/02/10.
  5. HTN, with poor medical control
  6. COPD
  7. NSVT*1
  8. PVC=15.8/Hrs
  9. HBV carrier
- 出院:Colon Cancer, c T1 N0 M0, Stage I
  - cos tattoo on 10/5
  - Laparoscopic right colectomy and anastomosis on 10/6.
  adenocarcinoma, pT1N0(0/17). MD.
  [Hx]
  1. HFrEF, FC III, LVEF:24%
  2. Complete AV block s/p PPM insertion on 1996/8/20 (DDDR mode), upgrade to Cardiac resynchronization therapy on 2010/3/5, CRT change generator on 2020/12/21. with generator in pending failure
  3. CAD-TVD s/p PCI + stent on 2013/9, 2013/10
  4. Moderate to severe AR, moderate MR, with severe impair LV function(LVEF 19%) s/p Mitral valve repair (posterior annuloplasty with SJM 31 mm ring) + Aortic valve replacement with tissue valve (SJM porcine valve 25 mm) on 2014/02/10.
  5. HTN, with poor medical control
  6. COPD
  7. NSVT*1
  8. PVC=15.8/Hrs
  9. HBV carrier

主訴

- Constipation for months.

病史

- The 85-year-old man with history of
  1. HFrEF, FC III, LVEF:24%
  2. Complete AV block s/p PPM insertion on 1996/8/20 (DDDR mode), upgrade to Cardiac resynchronization therapy on 2010/3/5, CRT change generator on 2020/12/21. with generator in pending failure
  3. CAD-TVD s/p PCI + stent on 2013/9, 2013/10
  4. Moderate to severe AR, moderate MR, with severe impair LV function(LVEF 19%) s/p Mitral valve repair (posterior annuloplasty with SJM 31 mm ring) + Aortic valve replacement with tissue valve (SJM porcine valve 25 mm) on 2014/02/10.
  5. HTN, with poor medical control
  6. COPD
  7. NSVT*1
  8. PVC=15.8/Hrs
  The patient was partially dependent on his family.

  He had constipation for months. He went to LMD for help first. Colonscopy biopsy showed T colon adenocarcinoma on 9/8. Pre-op tumor marker disclosed CA-199(41.76) and CEA(4.75). With the impression of Colon Cancer, c T1 N0 M0, Stage I , he was admitted for operation(Laparoscopic right colectomy and anastomosis). We would arrange pre-op survey including lung function, cardiosonography, coach and LGI scopy.

體檢發現

-
  - General appearance :
    - easy looking , conscious clear , without cardiopulmonary distress
    - Vital signs自動帶入date 體溫(BT) 脈搏(P) 呼吸(R) 血壓(BP) 身高(BH) 體重(BW) BMI
      20211004 36.6℃ 75次/分 18次/分 158/84mmHg 156.7cm 65.1kg 26.5
    - Vital signs: B.P. 158/8 mmHg , P.R. 75 /min , R.R. 18 /min , B.T. 36.8 ℃
  - HEENT :
    - Eyes: no anemic conjunctiva, no icteric sclera, normal EOM
    - ENT: no otorrhea, no rhinorrhea, no erythematous mucosa, no injected tonsil, no deformity
  - Neck: supple, freely movable, trachea midline,no deformity, no engorged jugular vein , no palpable thyroid or lymph node enlargement
  - Chest:
    - Inspection: Grossly normal, symmetric and fully expansion, no accessory muscle used, no operation scar, no spider angiomata, no gynecomastia
    - Palpation: no palpable mass or nodule, no subcutaneous emphysema, no tenderness
    - Ausculation: clear breathing sound
  - Heart:
    - Palpation: no thrill, no heave
    - Percussion: normal heart size
    - Ausculation: regular heart beat, normal S1, S2, no gallop, no murmur
  - Extremities: No deformity, full ranged of motion without limitation, no clubbing finger or nail cyanosis, no pitting edema, no ulcer or pigmentation, no obviou

  CRS CONDITION

  - Karnofsky performance status: (100) Normal, no complaints, no evidence of disease
  - PE of Abdomen:
  - __1. Inspection: flat
  - __2. Ausculation: normoactive
  - __3. Palapation: no tenderness
  - __4. Percussion: mild tympanic
  - CEA: 4.75 ng/ml
  - Imaging Studies:
  - Colonoscope report: pending
  - Sono , CT of abdomen and chest Films:檢驗項目 :CT ABDOMEN-SURVEY 報告日期 :1100928
    主訴：
    Result:N Finding: Post IV contrast CT scan of the whole abdomen with 600cc oral contrast showed: > A 3.3cm gallstone. > No obvious wall-thickening or mass lesion noted over the visible colon. > No obvious enlarged lymphadenopathy can be identified at paraaortic retroperitoneum or pelvis. > DJD change of T-L spine with spur formation. Impression: As above description.

- Figures:
    1. colon & rectum

手術日期及方法

- 手術日期及方法20211006 1. Laparoscopic right hemicolectomy with side-to-side anastomosis with EndoGIA 60mm*1 and handsewen technique

  2. Ileo-colic artery, right colic artery and middle colic artery was ligated via Hemo-clips.
  3. Take down of hepatic flexure : (+)
  4. Cut margin: proximal (ileum) >10 cm, (colon) 3 cm, distal 3 cm.
  5. Repair of mesentery defect : Nil.
  6. One 12 Fr. penrose drain was placed from RLQ area at Morrison's pouch.

住院治療經過

- After admission, pre-operation survey showed no contraindication for the operation. Thus, the operation(Laparoscopic right colectomy and anastomosis) was performed smoothly on 2021/10/06. The postoperative course was rather stable, we gradually titrate her diet from water to current warm I diet. There was no obstructive nor leakage sign. Lab data revealed hypokalemia and hypocalcemia, we prescribed instant electrolyte supplement. There was no muscle cramps, nor general malaise. Pathology discolosed tubular adenoma, LN(0/17),no residual malignancy. Under relatively stable status, he may be discharged on 10/14.

合併症

- nil.

一般檢查

- 累積報告 :

DATE	TIME	ALB	TP	BIL.T	BIL.D	ALKP	AST	ALT	LDH	AMY	LIPASE	NH3	Ferritin	UA
1101007	0540	3.2					19	8						
1101012	0909							3						
1101012	1615	3.8												

DATE	TIME	WBC	RBC	HGB	HCT	MCV	PLT	BLST	NEUT	BAND	SEG	LYM	MON	EOS	BAS
1101007	0536	11270	4.09	12.8	38.5	94.1	175		81.3			10.8	6.2	1.6	0.1
1101012	0920	11440		13.3			241		80.7			10.6	6.6	1.9	0.2

DATE	TIME	NA	K	CL	BUN	CREAT	eGFR	CA	Mg	P	CRP	ESR	PCT	D-Dimer	LACTATE
1101007	0540	137	3.7	106	11	1.01	74.44	7.5							
1101012	0909	135	3.3		12	0.90	85.04	6.9							

- 放射線報告 :檢驗項目 **:CT, LUNG** 報告日期 **:1101005**

  主　訴：
  Result:Y
  Finding:
  CT of chest with IV contrast enhancement.
  > history of T-colon cancer. Tumor survey.
  > S/P pacemaker placement
  > S/P Median sternotomy for aortic valve replacement. Dilated LV.
  > No abnormal lung nodule is noted. No enlarged mediastinal or hilar node.
  > some parechymal bands are noted.
  > two thin wall peribronchial lung cysts over RUL
  > calcifications at LAD, LCX
  > mild thickening of right extrapleural fat
  > a 3cm gallbladder stone
  > left renal cyst.
  > DJD change with spur formation of T spine.
  > A small sclerotic lesion over right 9th rib and T8, bone island is suspected.
  > No significant enlarged LNs over mediastinum.
  > No enlargement of bil. adrenal glands.
  Impression:
  No abnormal lung nodule is noted.
  No enlarged mediastinal or hilar node.

  檢驗項目 **:CT ABDOMEN-SURVEY** 報告日期 **:1100928**
  主　訴：
  Result:N
  Finding:
  Post IV contrast CT scan of the whole abdomen with 600cc oral
  contrast showed:
  > A 3.3cm gallstone.
  > No obvious wall-thickening or mass lesion noted over the visible colon.
  > No obvious enlarged lymphadenopathy can be identified at
  paraaortic retroperitoneum or pelvis.
  > DJD change of T-L spine with spur formation.
  Impression:
  As above description.
- nil.

特殊檢查

- PFT :PULMONARY LABORATORY F-V LOOP PRE-RX POST-RX SPIROMETRY (BTPS) PRED ACTUAL %PRED ACTUAL %PRED %CHG FVC LITERS 1.88 1.31 69 FEV1 LITERS 1.39 0.98 70 FEV1/FVC % N/A 75 FEF25-75% L/SEC 1.43 0.73 51 FEF25% L/SEC 5.13 2.24 44 FEF50% L/SEC 2.03 0.97 48 FEF75% L/SEC 0.40 0.24 61 FEF200-1200 L/SEC PEF L/SEC 5.73 3.37 59 FIVC LITTERS FIF50% L/SEC N/A 0.87 PIF L/SEC N/A 1.09 FEF50/FIF50 UNITLESS MVV 78.73

病理報告

- 活體組織切片病理報告　手術日期：**110/10/05**　病理號：**S1031793**

  診斷醫師:陳律臻 報告日期:110/10/12
  Pathologic diagnosis:
  Colon, 25 cm from anal verge, endoscopic biopsy --- Tubular adenoma.
  Prognostic and predictive factor:
  1. Degree of dysplasia: Low grade.
  2. Margin of stalk: Cannot be well evaluated.
  Gross description:
  The specimen consists of a piece of tan soft tissue, 0.5x0.3x0.3 cm.
  All for section.
  Microscopic description:
  Section shows colon mucosa with tubular glands proliferation with
  dysplasia.
  #T-59300_2 #M-82110_2 0 1340 000000

  活體組織切片病理報告　手術日期：**110/10/06**　病理號：**S1031951**

  診斷醫師:陳志榮 報告日期:110/10/13
  Pathologic diagnosis:
  Colon, terminal ileum, and appendix, right hemicolectomy --- a. S/P
  EMR without residual malignancy. b. Tubular adenoma. c. Unremarkable
  lymph node (0/17).
  Gross description:
  The specimen consists of a segment of colon, 19 cm in length, and
  3.5 cm in proximal circumference, 2 cm in distal cut end
  circumference, and a segment of terminal ileum, 5.5 cm in length, and
  2 cm in cut end circumference. A hematoma, 3.5x3x2 cm, is seen near
  the ileocecal valve. A clip is seen, cm to the distal cut end. On
  cross section, no defenite invasion is seen. A polyp, 0.2x0.2x0.2cm,
  is seen 4.8 cm to the ileocecal valve. The appendix, ileocecal valve,

- and both cut ends are free of tumor. Representative part for section: A,B) distal cut end and mucosa around the clip C) radial margin D) proximal cut end E) ileocecal valve F) polyp G-J) hematoma and appendix K-M) lymph nodes.
  #T-59420_2 #M-43000_2 0 1105 000000
  #T-C4560_2 #M-09183_2 0 1105 000000
  #T-59200_2 #M-09183_2 0 1105 000000

其它

- 檢驗項目 :**Colonoscopic + polypectomy** 報告日期 :**1101005**
  Result:
  年齡：
  內視鏡型號： 290-21 檢查開始時間： 1545 檢查至盲腸時間： 1555 檢查結束時間：
  1600
  ————————————————————————————————
  適應症(Indication)： 其他臨床目的施行之大腸鏡 PRE OP SURVEY
  大腸鏡伸入位置(Insertion level)最深位置： Cecum (距離肛門口 公分)
  未達盲腸原因：(複選) N N
  N N N
  檢查前用藥(Premedication)：
  止痙攣藥物：
  無
  止痛鎮靜藥物：
  N N N
  N N
  清腸用藥(Colon cleansing agent)：(複選)
  N N N N N N
  N
  清腸給藥時間(Preparation time)： Split dose
  清腸程度(Colon cleansing level)： 良好(Excellent)
  抗凝血藥物(Anti platelet/Coagulant)： 無
  Y N N
  大腸鏡檢後併發症(Complication)：(複選)
  N N
  檢體總數： 1 顆(含增生性瘜肉) 臨床診斷為C2(腺瘤)或C3(其他瘜肉)或D(腫瘤)應逐顆呈現
  若有2個以上診斷結果，填寫順序依序為4大腸癌＞3瘜肉＞2潰瘍性大腸炎＞
  1痔瘡＞5其他>0正常
  檢查結果(Findings)：
  大腸癌
  20211005 COLONOSCOPY WAS UP TO CECUM,
  1.TUMOR AT T-COLON, S/P TATTOO
  2.A COLON POLYP AT 25CM FAV, S/P COLD SNARE POLYPECTOMY.
  T-COLON TUMOR
  COLON POLYP
  診斷：
  其他：
  病灶處未處置原因：(複選)
  N
  N
  N
  N
  N N
  N
  N
  病灶未送病理原因：
  註1:(若病灶不止一個，請再自行增加欄位)。
  註2:若臨床診斷(內視鏡診斷)診斷為「增生性瘜肉」，得無須送驗檢體；惟，需算在總顆數內。
  註3:無病灶：如完全正常，至少應附8張照片(盲腸、升結腸、肝彎曲、橫結腸、脾彎曲、降結腸、乙狀結腸、直腸)；如有病灶應附病灶照片。(照片無須上傳，留於醫院存查)
  註4:清腸程度
  良好(Excellent)：僅有少量的清澈糞水，且95%以上的腸道黏膜可被清楚觀察
  適當(Good)：較多量的清澈糞水佔據小於25%的黏膜，且90%以上的腸道黏膜可被清楚觀察
  尚可(Fair)：半固體的糞便可被清除，清洗後90%以上的腸道黏膜可被清楚觀察
  不良(Poor)：半固體的糞便無法被清除，且不到90%的腸道黏膜可被清楚觀察
  註5:併發症Significant bleeding 瘜肉切除後發生出血情形：
  (1) 中度(Moderate):需輸血治療者。
  (2) 重度(Severe): 接受介入性治療者，例如：經血管攝影栓塞、外科手術等。
  ————————————————————————————————
  第1個病灶
  ————————————————————————————————
  臨床診斷(內視鏡診斷)： D腫瘤
  病灶處位置： 臨床診斷為C2(腺瘤)或C3(其他瘜肉)或D(腫瘤)，病灶處位置應為單選；其餘(非C2,C3,D)得複選
  N N N N N
  Y N N N
  N N N N N (距離肛門口 公分)
  病灶處處置：(複選)
  N N N N
  N
  N
  N N N
  N N Y N
  N N
  N N
  病灶處有無檢體： (無檢體以下無需填寫)
  無檢體
  檢體大小(內視鏡下大小)： 公分(小數點1位) 檢體編號： (以A到Z呈現，區分每一檢體)
  ————————————————————————————————
  第2個病灶
  ————————————————————————————————
  臨床診斷(內視鏡診斷)： C瘜肉
  病灶處位置： 臨床診斷為C2(腺瘤)或C3(其他瘜肉)或D(腫瘤)，病灶處位置應為單選；其餘(非C2,C3,D)得複選
  N N N N N
  N N N N
  N N N N N (距離肛門口 25 公分)
  病灶處處置：(複選)
  N N N N
  Y N
  N
  N N
  N N N
  N
  N
  N
  N N
  病灶處有無檢體：

- (無檢體以下無需填寫)
  有檢體
  檢體大小(內視鏡下大小)：
  公分(小數點1位) 檢體編號： A (以A到Z呈現，區分每 檢體)

第3個病灶

臨床診斷(內視鏡診斷)：
病灶處位置： 臨床診斷為C2(腺瘤)或C3(其他惡丸)或D(腫瘤)，病灶處位置應為單選；其餘(非C2,C3,D)得複選
N N N N
N
N N N N
N N N N N (距離肛門口 公分)
病灶處處置：(複選)
N N N N
N N
N N
N
N N
N N
N
N
N N
病灶處有無檢體：
(無檢體以下無需填寫)
檢體大小(內視鏡下大小)：
公分(小數點1位) 檢體編號： (以A到Z呈現，區分每一檢體)

第4個病灶

臨床診斷(內視鏡診斷)：
病灶處位置： 臨床診斷為C2(腺瘤)或C3(其他惡丸)或D(腫瘤)，病灶處位置應為單選；其餘(非C2,C3,D)得複選
N N N N N
N N N N
N N N N N (距離肛門口 公分)
病灶處處置：(複選)
N N N N
N N
N N
N
N
N N
N
N
N
N N
病灶處有無檢體： (無檢體以下無需填寫)
檢體大小(內視鏡下大小)： 公分(小數點1位) 檢體編號：
(以A到Z呈現，區分每一檢體)

第5個病灶

臨床診斷(內視鏡診斷)：
病灶處位置： 臨床診斷為C2(腺瘤)或C3(其他惡丸)或D(腫瘤)，病灶處位置應為單選；其餘(非C2,C3,D)得複選
N N N N N
N N N N
N N N N N (距離肛門口 公分)
病灶處處置：(複選)
N N N N
N N
N N
N
N
N N
N
N
N
N N
病灶處有無檢體： (無檢體以下無需填寫)
檢體大小(內視鏡下大小)： 公分(小數點1位) 檢體編號： (以A到Z呈現，區分每一檢體)

其他病灶

出院時情況

- 3.改門診治療

出院指示

藥名	劑量	劑量單位	用法	頻次	數量	數量單位	現況	天數	單位
Cal. Acetate 667mg	1	TAB	PO	TIDCC	15	TAB	TH	5	天
Voren-K tab 25mg	1	TAB	PO	BIDPRN	10	TAB	TH	5	天
Ultracet tab	1	TAB	PO	Q6H	20	TAB	TH	5	天

出院計劃

- 門診日期
  - date on 2021/10/18

主治醫師 2222H 林俊余 / 住院醫師 9727A 林順鴻

正式摘要(電子簽章：000000227293063)

W83 / W83

doctorRank:B
